# Fungal siderophore metabolism with a focus on *Aspergillus fumigatus*: impact on biotic interactions and potential translational applications

**DOI:** 10.1042/EBC20220252

**Published:** 2023-09-13

**Authors:** Isidor Happacher, Mario Aguiar, Annie Yap, Clemens Decristoforo, Hubertus Haas

**Affiliations:** 1 Institute of Molecular Biology, Biocenter, Medical University of Innsbruck, 6020 Innsbruck, Austria; 2 Department of Nuclear Medicine, Medical University Innsbruck, Innsbruck, Austria

**Keywords:** antifungal, diagnostics, fungi, iron, molecular imaging, siderophore

## Abstract

Iron is an essential trace element that is limiting in most habitats including hosts for fungal pathogens. Siderophores are iron-chelators synthesized by most fungal species for high-affinity uptake and intracellular handling of iron. Moreover, virtually all fungal species including those lacking siderophore biosynthesis appear to be able to utilize siderophores produced by other species. Siderophore biosynthesis has been shown to be crucial for virulence of several fungal pathogens infecting animals and plants revealing induction of this iron acquisition system during virulence, which offers translational potential of this fungal-specific system. The present article summarizes the current knowledge on the fungal siderophore system with a focus on *Aspergillus fumigatus* and its potential translational application including noninvasive diagnosis of fungal infections via urine samples, imaging of fungal infections via labeling of siderophores with radionuclides such as Gallium-68 for detection with positron emission tomography, conjugation of siderophores with fluorescent probes, and development of novel antifungal strategies.

## Introduction

**Figure 1:**
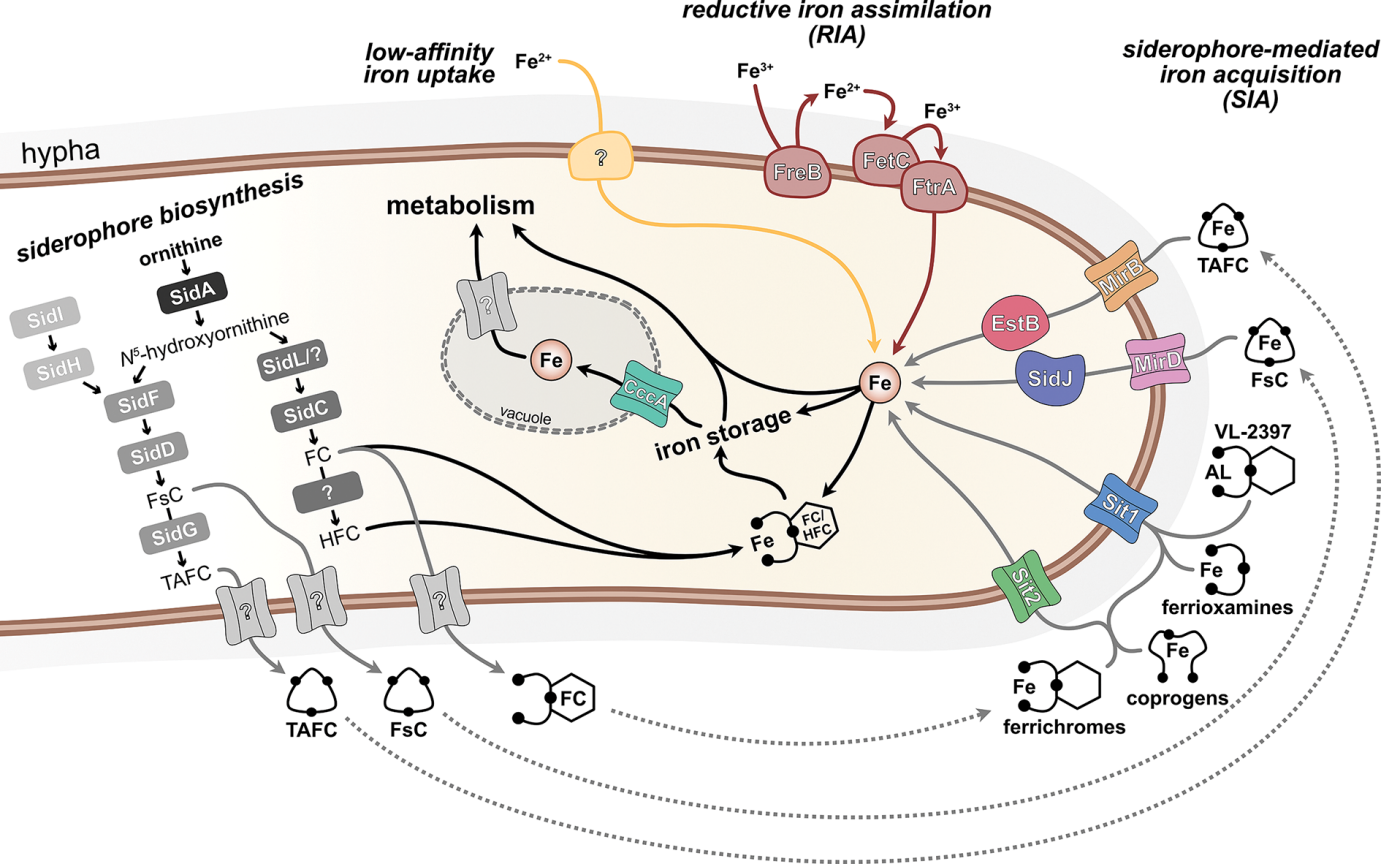
Schematic summary of iron acquisition and storage in *A.* *fumigatus* For iron uptake, *A. fumigatus* employs uncharacterized low-affinity and two high-affinity systems, RIA and SIA. RIA involves reduction of ferric iron by FreB, reoxidation of resulting ferrous iron by FetC and uptake of ferric iron by FtrA. Enzymatic steps in siderophore biosynthesis, described in detail in the text, are shown in gray. After secretion of the siderophores by yet uncharacterized mechanisms and chelation of ambient iron, siderophore-iron complexes are taken up into the cell by the substrate-specific SITs: MirB, MirD, Sit1, and Sit2. In addition to the endogenous siderophores FsC, TAFC, and FC, *A. fumigatus* is able to utilize several xenosiderophores including ferrioxamines and coprogens. FC not only is involved in iron uptake but also plays a role in storage and intra-/transcellular transport of iron; HFC is used for conidial iron storage. Excessive iron is detoxified by CccA-mediated transport into the vacuole. It is unknown, weather vacuolar stored iron can be reused. FsC and TAFC are hydrolyzed to release the iron by SidJ and EstB, respectively. Abbreviations: FC, ferricrocin; FsC, fusarinine C; HFC, hydroxyferricrocin; TAFC, triacetylfusarinine C; ?, uncharacterized or questionable mechanisms.

Fungi colonize an immense range of habitats involving mutualistic, competitive, and pathogenic relationships with other organisms [1–3]. Due to their diverse survival mechanisms and unique attributes, fungi thus play important roles in recycling of organic material, in food production, in biotechnology as producers of valuable primary and secondary metabolites, as well as in medicine and agriculture as producers of toxins and as pathogens for animals and plants. A central prerequisite for habitat colonization is uptake of nutrients including iron which is an essential trace element for all eukaryotes and nearly all prokaryotes. This metal is one of the most abundant elements on earth. However, its bioavailability is very low in most biological niches as it is easily oxidized by atmospheric oxygen into hardly soluble ferric complexes such as ferric hydroxides [4]. Furthermore, fungal pathogens of animals and plants are confronted with iron limitation during host infection [5]. On the other hand, excessive iron uptake is toxic as free iron causes oxidative stress [6,7]. Therefore, fungi have evolved different, highly controlled, high-affinity iron acquisition strategies including utilization of heme, reductive iron assimilation (RIA), and siderophore-mediated iron acquisition (SIA) [5,8]. For RIA, ferric iron is reduced by membrane-localized metalloreductases such as FreB and reoxidized and taken up by the FetC/FtrA protein complex in *Aspergillus fumigatus* [5]. Siderophores are low molecular mass (about 1 kD), ferric iron (Fe^3+^)-specific chelators employed by bacteria and fungi for iron acquisition and by fungi also for intracellular iron handling [5,9]. The presesnt review focuses on SIA and mainly on *A. fumigatus*, as SIA has been studied in most detail in this mold. *A. fumigatus* has been shown to employ RIA and SIA but appears to lack efficient use of heme as iron source [10]. Control of iron uptake and storage appears to be the major mechanism to maintain fungal iron homeostasis as no excretory mechanism for iron has been identified so far. Iron detoxification relies mainly on vacuolar iron deposition as shown in *Saccharomyces cerevisiae* and *A. fumigatus*; the transporter is termed CccA in *A. fumigatus*, whereby it is unknown if vacuolar stored iron can be reused in *A. fumigatus* [11]. The strategies for iron uptake and storage of *A. fumigatus* are summarized in Figure 1.

## Siderophore biosynthesis

**Figure 2:**
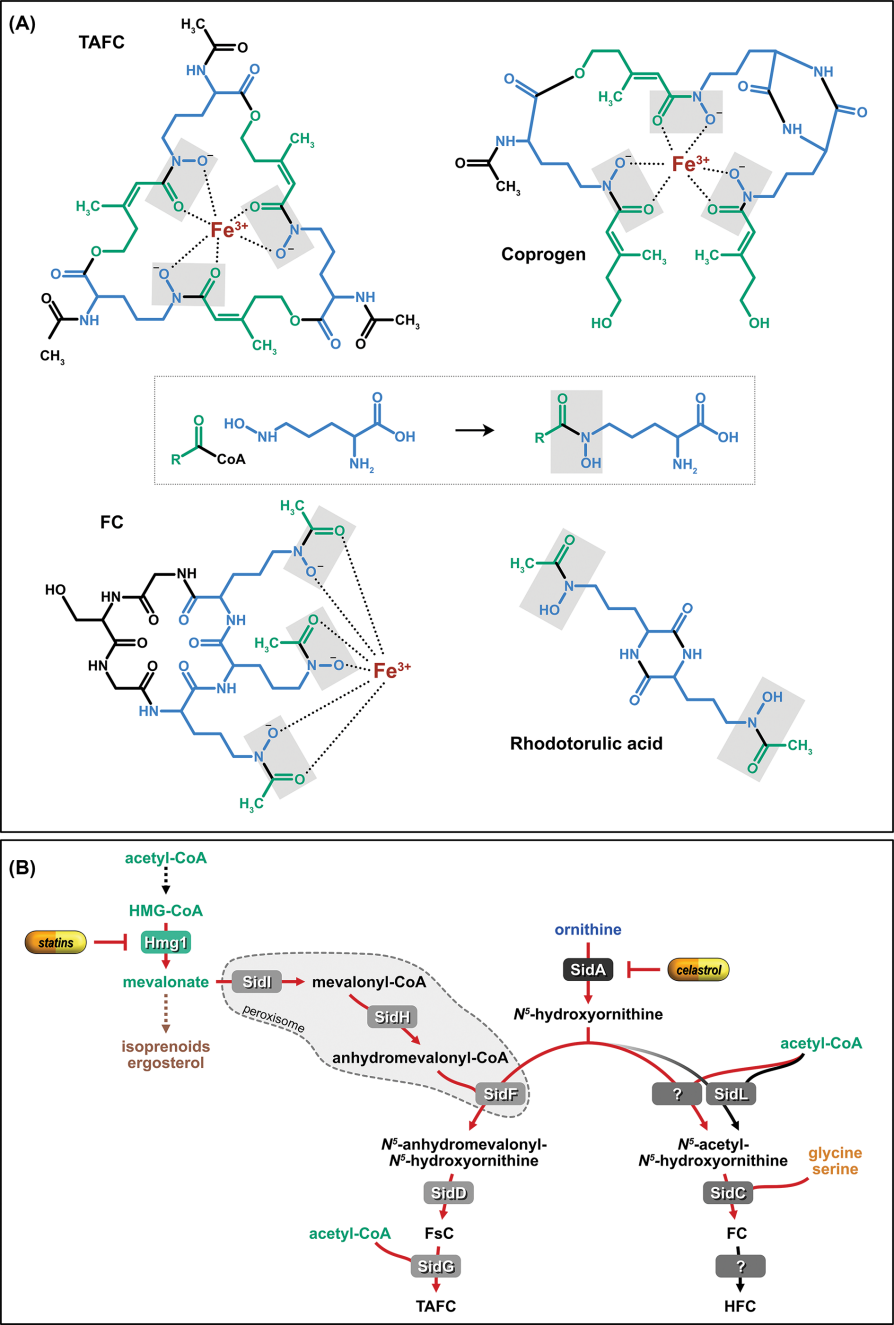
Structures of fungal hydroxamate-type siderophore representatives and the siderophore biosynthetic pathway of *A. fumigatus* (**A**) All hydroxamate-type siderophores share common building blocks, *N^5^*-hydroxyornithine (in blue) and acyl/acetyl groups (in green), which together constitute the hydroxamate-groups (boxed in gray; middle panel). Hydroxamate groups are shaded in gray. In FsC, TAFC, and coprogen, the acyl group is anhydromevalonyl; in rhodotorulic acid and FC, it is acetyl. The acetyl groups lacking in FsC compared with TAFC are shown in black. (**B**) The siderophore biosynthetic pathway is described in detail in the text. Enzymes exclusively dedicated to siderophore biosynthesis are shaded in gray; siderophore pathway intermediates are in black; red arrows mark steps that are transcriptionally up-regulated by iron limitation. This figure was reproduced in part from Figure 2 of [5].

Most *Ascomycota* and *Basidiomycota* species produce hydroxamate-type siderophores displaying a remarkable species-specific structural variety [5]. However, there are prominent exceptions lacking siderophore biosynthesis such as the entire *Saccharomycotina* clade, including *Saccharomyces cerevisiae* and *Candida albicans*, as well as the basidiomycete genus *Cryptococcus* spp. [5]. *Mucoromycota* lack hydroxmate-type siderophores but produce a carboxylate-type siderophore, termed rhizoferrin, originally isolated from *Rhizopus microsporus* [12,13]. Rhizoferrin displays a significantly lower affinity to iron compared with hydroxamates [12]. Rhizoferrin biosynthesis has been shown to depend on a nonribosomal peptide synthetases (NRPS)-independent synthetase (NIS) in *Rhizopus delemar* [14]. Apart from that, little is known about rhizoferrin-mediated iron uptake. Fungal hydroxamate-type siderophores are grouped into four structural distinct types: fusarinines, coprogens, ferrichromes, and rhodotorulic acid; representatives of each family are shown in Figure 2A. Notably, the terms ferrichrome and coprogen refer to specific siderophores in their structural type. Unfortunately, the siderophore name refers in some cases to the iron-complexed form (e.g., ferrichrome) and in some cases to the metal-free form (e.g., coprogen). Siderophores are synthesized in the metal-free form but taken up only as metal complex. Detailed chemistry of the structural diversity of fungal siderophores has been reviewed previously [9,15]. In addition to hydroxamate- and carboxylate-type siderophores, bacteria produce also catecholate-, carboxylate-, and mixed-type siderophores [9,16]. Plants employ rather simple iron chelators such as nicotianamine, mugineic acid family phytosiderophores, and citrate for iron mobilization [17].

**Figure 3:**
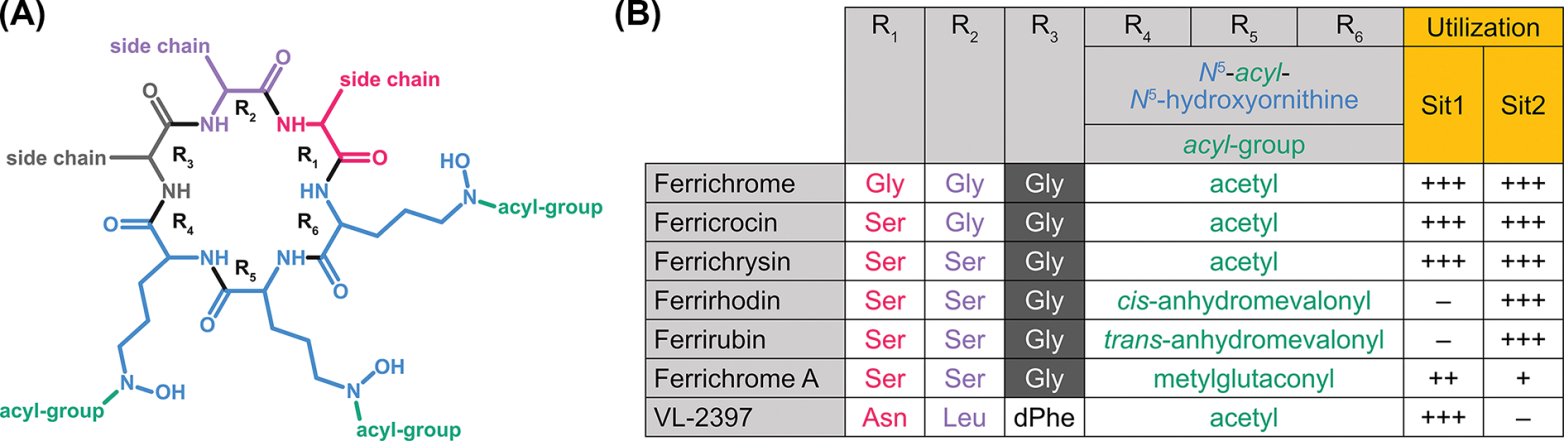
Impact of ferrichrome type on recognition by *A. fumigatus* Sit1 and Sit2 (**A**) Scheme of ferrichrome-type siderophore compositions with positions R_1_–R_6_. (**B**) Comparison of the ferrichrome-type siderophores with respect to composition and utilization. These ferrichrome-types differ in the *N^5^*-acyl groups present in positions R_4_–R_6_ and the amino acid residues present in positions R_1_–R_3_, whereby glycine (shaded in dark gray) is present in position R_3_ in all these ferrichrome-types except VL-2397. Degree of utilization is marked by +++ high, ++ weak, + very weak, and − no utilization. This figure was reproduced in part from Figure 4 of [19].

Siderophore biosynthesis has been studied in most detail in *A. fumigatus* (Figure 2B). This mold secretes two fusarinine-type siderophores, fusarinine C (FsC), and triacetylfusarinine C (TAFC) to capture environmental iron [18,19]. Moreover, it employs two ferrichrome-type siderophores, hyphal ferricrocin (FC), and conidial hydroxyferricrocin (HFC) for intracellular handling of iron such as transport of iron through conidiophores for conidiation and conidial iron storage [18,20,21]. A recent study indicated that FC is also secreted and plays a particular role for iron uptake during germination [22]. The common initial step for biosynthesis of all hydroxamate-type siderophores is formation of *N^5^*-hydroxyornithine from ornithine, which is catalyzed by the monooxygenase SidA [10]. Subsequently, the pathways for biosynthesis of different hydroxamate types split due to incorporation of different acyl-groups, which generates the iron-chelating hydroxamate group. For biosynthesis of fusarinine- and coprogen-type siderophores, the transacylase SidF transfers anhydromevalonyl-CoA to *N^5^*-hydroxyornithine [18,23,24]. Anhydromevalonyl-CoA is derived from mevalonate by the mevalonyl-CoA ligase SidI and the mevalonyl-CoA hydratase SidH [18,23,24]. SidI, SidH, and SidF are localized in peroxisomes, while the other siderophore biosynthetic enzymes are assumed to function in the cytosol [25]. The linkage of three *N^5^*-anhydromevalonyl-*N^5^*-hydroxyornithine to cyclic FsC or linear coprogens is mediated by NRPSs displaying a similar architecture, e.g., *A. fumigatus* SidD for FsC and *Cochliobolus heterostrophus* Nps6 for coprogens [18,26,27]. FsC is then triacetylated by SidG to form more stable TAFC [18]. Derivatization of coprogen B, including acetylation and methylation, allows for the diversity of coprogen-type siderophores. For synthesis of ferrichrome-type siderophores, which are cyclic hexapeptides, *N^5^*-hydroxyornithine is acylated, e.g., in *A. fumigatus* acetylated to *N^5^*-acetyl-*N^5^*-hydroxyornithine by the transacetylase SidL and another yet unknown enzyme [28]. Subsequently, the hexapeptide FC is assembled by the NRPS SidC from three *N^5^*-acetyl-*N^5^*-hydroxyornithine, two glycine, and one serine residue [5,24]. Conidial HFC is formed from FC by a single hydroxylation by an as yet unknown enzyme [18]. Figure 3 displays some ferrichrome-type siderophores containing different amino acids and acyl-groups. Notably, Epichloënin A produced by *Epichloë festuce* is an atypical ferrichrome-type siderophore consisting of eight amino acid residues [29]. The presence of three hydroxamate groups, as found in most hydroxamate-type siderophores, allows formation of hexadentate structures, which provide the highest affinity for ferric iron [9]. The mechanism of cellular siderophore export has not been characterized yet.

## Siderophore uptake

Uptake of hydroxamate-type siderophore-iron chelates in fungi is mediated by members of the siderophore iron transporter (SIT) family, which is a subfamily of the major facilitator superfamily. SITs are exclusively found in the fungal kingdom. Most species from all fungal phyla (*Ascomycota*, *Basidiomycota*, *Mucoromycota*, and *Chytridiomycota*) possess SITs [5,30]. Remarkably, species that lack siderophore production have preserved the ability to utilize siderophores. The ability to utilize xenosiderophores (siderophore-types other than self-produced ones) saves energy and most likely plays a role in microbial interaction (see below).

Functional characterization of SITs started in *S. cerevisiae*, which possesses four SITs with different substrate specificity: Sit1p/Arn3p for ferrioxamine B (a bacterial hydroxamate-type siderophore), Arn1p for ferrichromes, Taf1p/Arn2p for TAFC, Enb1p/Arn4p for the bacterial catecholate-type siderophore enterobactin [31]. In contrast, *C. albicans* contains only a single broad-range substrate-specific SIT, CaArn1p/CaSit1p [31]. Phylogenetic analysis revealed that all *Saccharomycotina* SITs are more similar to each other than they are to SITs from other fungal species [19,30], which indicates that these transporters arose after the divergence from the other species. Consequently, the substrate specificity of *non-Saccharomycotina* SITs, such as those of mold species, cannot be predicted on the basis of sequence similarity to *S*. *cerevisiae* SITs.

*A. fumigatus* can utilize a variety of hydroxamate-type siderophores, including several ferrichromes, endogenously secreted FsC and TAFC, several ferrioxamines, and coprogens, although the latter are utilized only poorly [19]. In contrast, *A. fumigatus* was found to be unable to utilize the hydroxamate-type siderophores basidiochrome and rhodotorulic acid, the bacterial catecholate-type siderophore enterobactin, rhizoferrin, and the mixed-type siderophores ornibactin and schizokinen [19]. Recent studies revealed the substrate specificities of four SITs (Sit1, Sit2, MirB, MirD) of *A. fumigatus* [19,32,33]. Sit1 and Sit2 were found to be essential for the utilization of coprogen- and ferrichrome-type siderophores, displaying both redundancy and exclusivity depending on the amino acid residues and acyl-groups present in the respective hexapeptide [19], which is summarized in Figure 3. In detail, (i) both Sit1 and Sit2 accept serine and glycine in positions R_1_ and R_2_ and acetyl as acyl-group in R_4_–R_6_; (ii) Sit2 but not Sit1 accepts anhydromevalonyl as acyl-group in positions R_4_–R_6_; (iii) Sit2 does not distinguish between *cis-* and *trans-*anhydromevalonyl as acyl-group in positions R_4_–R_6_; (iv) Sit1, and to a lesser extent Sit2, accept methylglutaconyl as acyl-group in positions R_4_–R_6_; (v) methylglutaconyl as acyl-group in R_4_–R_6_ significantly decreases uptake efficacy in comparison with anhydromevalonyl; and (vi) Sit1 accepts asparagine, leucine, and D-phenylalanine in positions R_1_, R_2_, and R_3_, while at least one of these amino acid residues disturbs recognition by Sit2 and therefore uptake of the ferrichrome-type antifungal VL-2397 depends exclusively on Sit1 [19,34]. These results demonstrate that both the amino acid residues in positions R_1_–R_3_ as well as the acyl-groups in positions R_4_–R_6_ impact recognition of ferrichrome-type siderophores. Similar to ferrichrome A, the utilization efficacy of coprogen-type siderophores was low despite the fact that these siderophores were accepted by both Sit1 and Sit2 [19]. Furthermore, Sit1 was shown to be the exclusive transporter of bacterial ferrioxamines [19], and that the transport efficacy of linear ferrioxamines is impacted by their charge and consequently the environmental pH [35]. Acquisition of TAFC was found to depend exclusively on MirB and that of FsC mainly on MirD [33]. Taken together, for every siderophore known to be utilized by *A. fumigatus*, a corresponding major transporter has been identified. Sit1, Sit2, and MirD were found to be dispensable for virulence in murine models of pulmonary aspergillosis [19,32,33]. In line with TAFC being the major secreted siderophore for iron acquisition, MirB is important for virulence [33]. The loss of MirB not only impaired uptake of TAFC but additionally caused an autoinhibitory effect by decreasing the bioavailability of environmental iron due to chelation by a now futile siderophore. Moreover, the *Aspergillus nidulans* SIT MirA was indicated to transport bacterial catecholate-type siderophore enterobactin [36]. The insights in the substrate specificity of SITs will help to reveal the molecular basis of substrate recognition.

**Figure 4:**
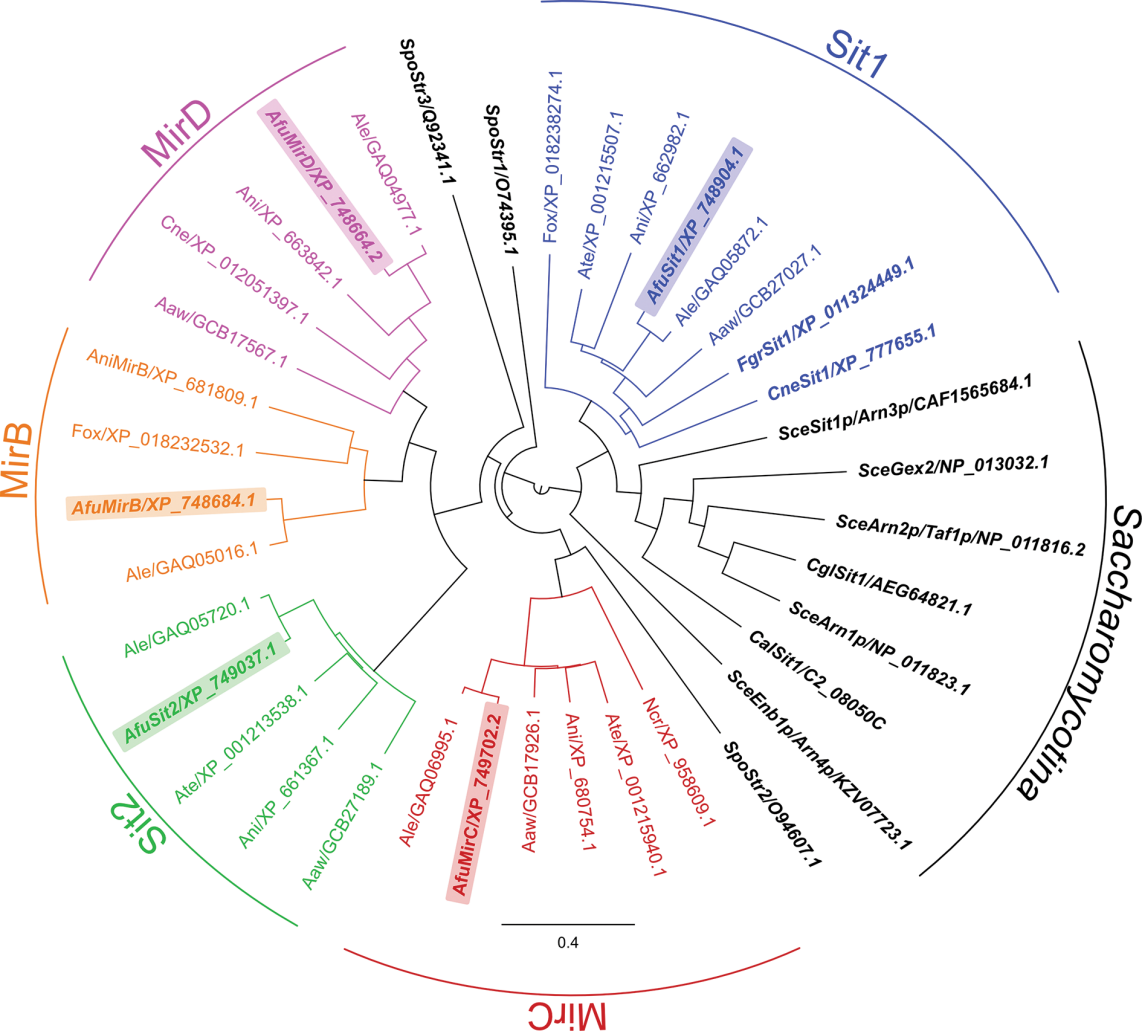
Phylogenetic analysis conducted with 38 SITs from 13 fungal species * Aspergillus fumigatus* (Afu), *Aspergillus nidulans* (Ani), *Aspergillus lentulus* (Ale), *Aspergillus awamori* (Aaw), *Aspergillus terreus* (Ate), *Fusarium oxysporum* (Fox), *Fusarium graminearum* (Fgr), *Schizosaccharomyces pombe* (Spo), *Saccharomyces cerevisiae* (Sce), *Neurospora crassa* (Ncr), *Cryptococcus neoformans* (Cne), *Candida albicans* (Cal), and *Candida glabrata* (Cgl). Scale bar shows the percentage of genetic variation. SITs with identified substrates are in italics, SITs from *A. fumigatus* are shaded in different colors. Data for this figure were taken from Figure 3 of [19].

Phylogenetic analysis of 38 SITs from 13 fungal species shown in Figure 4 demonstrated that the four functionally characterized *A. fumigatus* SITs belong to different subclades [19]. As mentioned above, all SIT family members of the *Saccharomycotina* species *S. cerevisiae*, *C. albicans*, and *C. glabrata* are closely related building a sister clade with the ‘Sit1 clade,’ which indicates a common origin. Within the *A. fumigatus* Sit1 subclade, *Fusarium graminearum* Sit1 has been shown to transport ferrichrome and ferrioxamine B and *Cryptococcus neoformans* Sit1 was found to transport ferrioxamine B [37,38], which underlines a link between phylogenetic clustering substrate specificity. Despite overlapping substrate specificities, Sit1 and Sit2 are only distantly related, while MirB and MirD are localized in sister clades, indicating coevolution. The latter finding might be related to the fact that TAFC is biochemically derived from FsC, requiring only a single enzymatic step, i.e., triacetylation catalyzed by SidG [18]. Among the species analyzed, MirB was found to be conserved in *Fusarium oxysporum, Aspergillus lentulus* and *A. nidulans*. Importantly, the *A. nidulan*s MirB homolog was shown to transport TAFC when heterologously expressed in *S. cerevisiae* [36]. Another subclade in the phylogenetic analysis comprises homologs of a fifth putative siderophore transporter of *A. fumigatus*, MirC, which might play a function in FC biosynthesis [39]. Notably, two SITs shown in the phylogenetic analysis have been shown to accept nonsiderophore substrates; as *S. cerevisiae* Gex2 and *Schizosaccharomyces pombe* Str3 have been reported to transport glutathione and heme, respectively [40,41]. In contrast, *S. pombe* Str1 was found to transport ferrichrome [42]. Taken together, these data indicate the value of phylogenetic analysis for prediction of SIT substrate specificities outside of the *Saccharomycotina* subclade.

## Intracellular iron release from siderophores

Due to the high affinity of siderophores to iron, intracellular mechanisms to release the chelated iron from siderophores are required. Three esterases have been identified, which intracellularly hydrolyze siderophores with narrow substrate specificity: *A. fumigatus* EstB for TAFC, *A. fumigatus* SidJ for FsC, and *A. nidulans* EstA for enterobactin [43–45]. In bacteria, iron is liberated from the ferric siderophore complex through reduction to ferrous iron by enzymes termed ferric reductases [46]. Such a mechanism has not been identified in the fungal kingdom yet.

## Regulation of SIA

Similar to other high-affinity iron acquisition mechanisms, SIA is induced by iron limitation and repressed by iron to avoid excessive iron uptake. *A. fumigatus* employs two iron-sensing transcription factors, termed SreA and HapX [5,47]. SreA harbors two Cys_2_Cys_2_ GATA-type zinc fingers, which recognize the consensus DNA sequence ATCWGATAA, separated by a cysteine-rich region (CRR), which most likely mediates iron sensing. HapX comprises several phylogenetically conserved domains: (i) a bZIP-type DNA-binding domain, (ii) a Hap4-like domain (Hap4L) for physical interaction with the CCAAT-binding complex (CBC, termed Hap complex in *S. cerevisiae*), and (iii) four CRR, whereby two CRR (CRR-A and CRR-B) have been implicated in iron sensing. During iron sufficiency, SreA transcriptionally represses high-affinity iron uptake, including RIA and SIA. During iron starvation, HapX represses iron-consuming pathways such as respiration, heme biosynthesis, TCA cycle, and vacuolar iron deposition to spare iron. Furthermore, HapX transcriptionally activates SIA [48]. Remarkably, iron excess converts HapX into an activator of iron-dependent pathways, particularly vacuolar iron deposition. Inactivation of both HapX and SreA is synthetically lethal, underlining the critical role of iron homeostasis in cellular survival [48,49]. Both HapX and SreA are highly conserved in *Ascomycota* and *Basidiomycota* species [5,47]. Interestingly, *Saccharomycetaceae*, including *S. cerevisae* but not *Candida albicans*, lost SreA, conserved only the iron detoxification function of HapX leading to Yap5 proteins, and evolved novel iron regulators termed Aft1/2 [5,47]. Iron sensing in *A. fumigatus* was shown to depend on mitochondrial but not cytosolic iron sulfur cluster biosynthesis [50]. Most likely SreA and HapX sense the cellular iron status via binding of [2Fe-2S] cluster with their CRR additionally involving the [2Fe-2S] cluster chaperon GrxD and glutathione [5,47,50].

Recent studies indicated that iron shortage is additionally sensed via iron-dependent metabolic pathways. Biosynthesis of the branched-chain amino acids leucine, isoleucine, and valine includes enzymes with iron–sulfur clusters as cofactors and consequently iron limitation leads to accumulation of the pathway intermediate α-isopropylmalate, which post-translationally activates the Zn_2_Cys_6_-type transcription factor LeuB for feedback activation of leucine biosynthesis [51]. Recently, LeuB was shown to be required for full transcriptional activation of SIA in *A. fumigatus* indicating integration of metabolic signals in control of iron homeostasis [52,53]. Moreover, the sterol regulatory element-binding protein (SREBP) transcription factor SrbA and the Gal4-type zinc finger protein AtrR were found to mediate activation of high-affinity iron acquisition including RIA and SIA [54,55]. Previously, SrbA and AtrR were found to be essential for sterol-feedback regulation and consequently resistance against triazole drugs as well as for hypoxic growth [56,57]. Consequently, SrbA and AtrR link control of iron acquisition and iron consumption as ergosterol biosynthesis and adaptation to oxygen limitation are iron-dependent pathways [23,58].

## The role of SIA in biotic interactions

Iron is an essential trace element that is limiting in most habitats including plant and animal hosts of fungal pathogens. Moreover, mammalian innate immunity further increases restriction of iron availability to fight infections within ‘nutritional immunity,’ leading to anemia of inflammation [59,60]. Consequently, pathogens evolved strategies to ‘steal’ the iron from their hosts. Thus, competition for iron is a critical battleground that determines the outcome of host–pathogen relationships. In agreement, iron overload decreases mammalian resistance against infection including invasive aspergillosis [61,62]. In healthy individuals, plasma iron accessible for cells is bound to the iron-transporting protein transferrin, whereby transferrin is typically only about 30% iron-saturated. However, pathologic iron excess conditions can exceed the binding capacity of transferrin leading to nontransferrin-bound iron (NTBI), which was found to stimulate the *in vitro* growth of *A. fumigatus* in serum from hematopoietic stem cell-transplanted patients [63]. The first link between siderophores and virulence was the association between infection with *Mucoromycota* species in dialysis patients and the use of desferrioxamine against aluminum overload and/or iron excess, a link that was confirmed in animal models in 1989 [64]. Later on, desferrioxamine was shown to serve as xenosiderophore for these species [65]. However, the finding that siderophore biosynthesis is dispensable for virulence of the maize pathogen *Ustilago maydis* in 1993 largely diminished the interest in fungal SIA [66]. This view changed significantly, when blocking siderophore biosynthesis by inactivation of SidA was found to render *A. fumigatus* avirulent in murine models for invasive aspergillosis in 2004, while blocking RIA had no effect [10]. Subsequently, biosynthesis of both fusarinine-type and ferrichrome-type siderophores and uptake of TAFC by MirB were found to be crucial for virulence of this mold [18,33]. Siderophore biosynthesis was also found to be crucial for virulence of several plant pathogenic species including *F. graminearum, C. heterostrophus*, and *Alternaria brassicicola* [26,67–69]. These fungal species are necrotrophs, which rapidly kill plant tissue after invasion. In contrast, *U. maydis* is a biotrophic pathogen that establishes a long-term feeding relationship with its hosts without causing immediate damage. Hemibiotrophs switch after a short initial biotrophic phase to necrotrophy. In the hemibiotrophic plant pathogen *Colletotrichum graminicola*, SIA was found to be repressed during biotrophic- and activated during necrotrophic growth [70]. In line with the importance of SIA for necrotrophic virulence and RIA for biotrophic virulence, both SIA and RIA were found to be crucial for virulence of *C. graminicola* but only RIA for virulence of *U. maydis* [71]. The presence of siderophores modulates the plant immune response and down-regulation of SIA might serve to evade the plant immune response in biotrophic pathogens [72–74]. Recently, the carboxylate-type siderophore rhizoferrin was shown to also play a role in virulence of the *Mucoromycota* species *Mucor lusitanicus* [75].

In line with the importance of SIA for virulence of *A. fumigatus*, metabolic pathways that are required in a nonexclusive manner for SIA were found to be crucial for virulence of *A. fumigatus* including mitochondrial production of the siderophore precursor ornithine [76], leucine biosynthetic enzymes that control post-translational activation of LeuB and consequently affect iron regulation [53], biosynthesis of riboflavin that is an essential cofactor for the first step of siderophore biosynthesis [77], and biosynthesis of pantothenic acid, which is a precursor of the substrate-binding 4’-phosphopantethine groups of NRPSs including SidD and SidC [77]. Lack of extracellular siderophore biosynthesis or SreA resulted in perturbation of the mutualistic interaction of the endophyte *E. festucae* and its perennial ryegrass host [29,78], which indicates that siderophores also play a role in symbiotic interactions.

The battle for iron significantly also impacts the interaction of microorganisms leading to beneficial and antagonistic relationships. What is the reason for the stunning structural diversity of siderophores produced by fungi and bacteria? Most likely, the rational is that siderophores produced by a species can either promote or repress the growth of another species in the same habitat depending on its siderophore utilization capacity: growth promotion by improving iron uptake in case of uptake of the siderophore and growth repression by chelation of iron by a siderophore that is not recognized. The optimal competitive strategy would therefore be the production of siderophores that are not recognized by competitors combined with the capacity to utilize as many siderophore structures as possible. This is the most likely explanation for the utilization of xenosiderophores also by siderophore-producing species, e.g., utilization of enterobactin via MirA by *A. nidulans* [36] or the utilization of ferrichrome- and coprogen-type siderophores, which are produced exclusively by fungal species, by bacteria such as *Escherichia coli* or *Salmonella* spp. [79–81]. Similarly, the utilization of siderophores by species that do not produce siderophores such as *Saccharomycotina* or *C. neoformans* might be explained by the potential antagonistic activity of siderophores or alternatively to save energy as siderophore-chelated iron represents a soluble energized iron-form. The interplay between *A. fumigatus* and the bacterium *Pseudomonas aeruginosa*, which frequently coinfects the airways of cystic fibrosis patients, visualizes the antagonistic activity of siderophores: *P. aeruginosa* can iron-starve *A. fumigatus* via iron sequestration by its major siderophore pyoverdine, which cannot be utilized by *A. fumigatus* [82]. In this competition for iron, siderophore production by *A. fumigatus* plays an important role in protection against *P. aeruginosa* [83]. In this respect, it is noteworthy that TAFC was found to inhibit growth of a range of bacterial species, which are obviously not able to utilize this siderophore [84]. The existence of natural siderophore-auxotrophic fungal species demonstrates the beneficial activity of siderophores. Examples are the mucoromycete *Pilobolus* spp., which requires coprogen or ferrichrome as growth factor [85], the ascomycete *Debaryomyces mycophilus*, which lives as endosymbiont in the guts of woodlice, and the basidiomycete *Tritirachium egenum*, which is a mycosymbiont lacking independent high-affinity iron acquisition and depending on xenosiderophore supply during growth in association with *Penicillium rugulosum* [86,87].

## Translational aspects of the fungal SIA

The use of siderophores distinguishes microbial from plant and mammalian cells, which might enable translational applications. The most prominent translational application of siderophores is the bacterial siderophore desferrioxamine B (trade name desferal®), which has been used in clinics for metal chelation therapy in iron or aluminum overload pathologies [88]. However, this therapy led to major problems with patient compliance due to the requirement for long periods of subcutaneous infusions of this orally ineffective drug. Consequently, desferal® was widely replaced by the orally applicable synthetic chelator drug deferasirox [88]. As most siderophores have a high affinity not only for iron but also for some other metals, desferrioxamine B was used as a chelator for radionuclides, initially for Gallium-67 for Single Photon Emission Tomography (SPECT) applications [89]. In recent years, desferrioxamine B has become the chelator of choice to label in particular antibodies with Zirkonium-89 (^89^Zr) for Positron Emission Tomography (PET), so-called immuno-PET imaging [90]. The reported *in vivo* release of ^89^Zr from the desferrioxamine B chelator has stimulated research to develop more stable chelators for PET imaging applications [91]. Among the developments, the use of FsC as scaffold to attach targeting vectors such as peptides has shown promising results to construct multimeric targeting probes for Gallium-68 (^68^Ga) and ^89^Zr labeling [92–94], as well as ^68^Ga-labeled probes for hybrid imaging [95] and pretargeting [96] applications. Using diacetylfusarinine C (TAFC lacking a single acetyl group allowing conjugation with the amino group) as bifunctional chelator to label an EGFR-targeting ZEGFR:2377 affibody with ^89^Zr revealed advantages over desferrioxamine B [97].

*A. fumigatus* is the most common human mold pathogen [98]. Invasive infections, occurring mainly in immunosuppressed patients, are rather rare but nevertheless life-threatening [98]. The diagnosis of fungal infections is difficult as it lacks specificity and sensitivity. Recently, TAFC was reported to be an attractive novel biomarker for systemic *A. fumigatus* infection enabling noninvasive diagnosis in human urine and bronchoalveolar lavage [99,100]. Detection of TAFC in urine is more sensitive than in serum because in murine and rat models, TAFC showed a short half-life in blood due to rapid renal excretion in intact form: within 45 min, about 90% of injected TAFC was found in kidneys and bladder [100]. In an *A. fumigatus* rat infection model not only TAFC but also FC was detected in urine and FC also in serum [101], which appears to have a longer half-life in serum compared with TAFC [102]. However, sensitive siderophore detection in human and animal samples is based so far on mass spectrometry, which represents a problem in clinics. This might change with optimization of detection with Raman spectroscopy or immunological assays employing a recently developed antibody recognizing TAFC [103,104].

**Figure 5:**
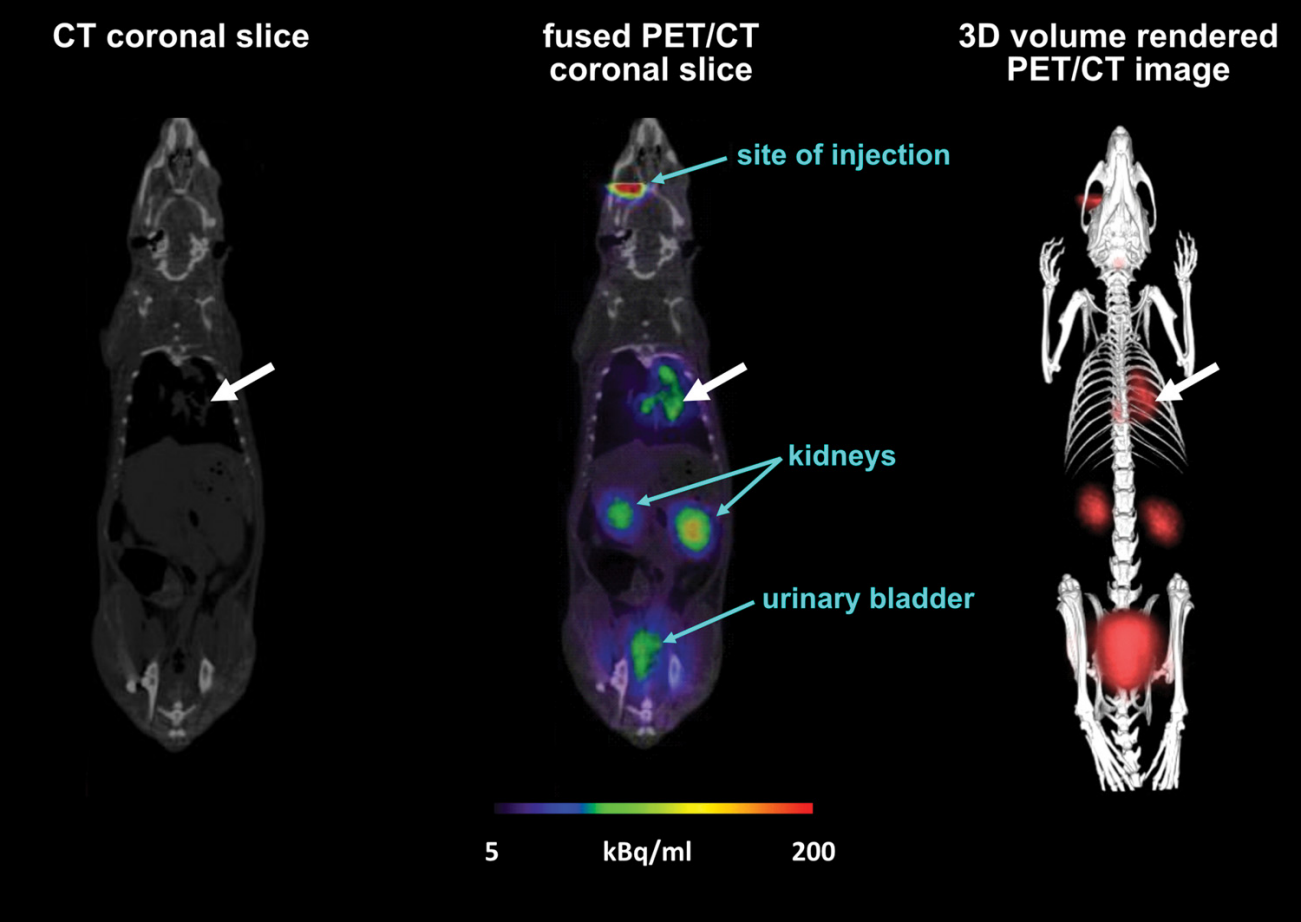
Static PET/CT images of *A. fumigatus* infected Lewis rats 45 min after injection of ^68^Ga–desferrioxamine B, indicating accumulation of the radiotracer in the pulmonary infection site (white arrow) as well as its renal excretion This figure was modified from Figure 2 of [35].

Furthermore, replacing iron in siderophores such as TAFC, ferrioxamine E, and ferrioxamine B by the radionuclide ^68^Ga allowed *in vivo* imaging of *A. fumigatus* infection by PET due to the specific uptake and accumulation of the siderophore in fungal cells [35,105,106], providing high specificity for detecting fungal infections [107]. Figure 5 shows PET/computer tomography (CT) images using ^68^Ga–desferrioxamine B for imaging pulmonary aspergillosis in a rat model [35]. Moreover, the conjugation of siderophores and fluorescent dyes enabled the generation of hybrid-imaging compounds, allowing the combination of PET and optical imaging in preclinical aspergillosis models [108]. Remarkably, conjugates of fluorescent dyes and diacetylfusarinine C showed subcellular localization dependent on the fluorescent molecule, i.e., conjugates with nitrobenzoxadiazole (NBD) and Ocean Blue accumulated in vacuoles, conjugates with BODIPY, silicon-rhodamine (SiR), and cyanine 5-carboxylic acid (Cy5) localized to mitochondria, and the conjugate with fluorescein isothiocyanate (FITC) showed cytoplasmic distribution [109]. These results emphasize that interpretation of the cellular fate of siderophores after uptake is problematic based on fluorescent labeling. Nevertheless, the uptake of all these compounds depended on the SIT MirB. These data revealed that SITs tolerate substantial derivatization of their substrate. This is particularly interesting as SITs represent one of few protein families that are unique to the fungal kingdom, i.e., they are not present in prokaryotes or other eukaryotes. Consequently, SITs might allow fungal-specific drug delivery by a Trojan horse approach [110,111], in which toxic compounds are conjugated to siderophores for selective import into fungal cells. TAFC derivatives appear most promising as the importance of MirB in virulence and the autoinhibition caused by its inactivation restrains its mutational inactivation and, consequently, the development of resistance at the level of uptake [33]. Synthesis of such derivatives resulted in compounds with traceable antifungal activities and accumulation in infected as shown by ^68^Ga-labeling and PET [112]. The natural ferrichrome-type antifungal drug VL-2397 (previously termed ASP2397), produced by *Acremonium persicinum* and containing aluminum instead of iron, was recently shown to require the SIT Sit1 for uptake and activity against *A. fumigatus*, while its mode of action remains elusive [34]. Notably, there are natural antibacterial siderophore-antibiotic conjugates, termed sideromycins and recently the first synthetic siderophore-antibiotic conjugate, cefiderocol, was FDA-approved to combat multidrug-resistant Gram-negative bacteria [113].

Due to its crucial role in virulence, the siderophore biosynthetic pathway represents a potential target for selective therapeutic intervention. A natural quinone methide, celastrol, was identified as a noncompetitive inhibitor of SidA [114]. Moreover, treatment of fungal keratitis in a murine model by dual topical therapy with the iron chelator deferiprone and statins, which blocks hydroxymethylglutaryl (HMG)-CoA reductase and consequently biosynthesis of isoprenoids and extracellular siderophores (Figure 2B), showed restriction of fungal growth [115].

## Summary

- Siderophores play a central role in maintenance of iron homeostasis in most fungal species.
- Siderophores display a stunning species-specific structural variety.
- Siderophores play a crucial role mutualistic and antagonistic biotic interactions including fungal virulence in animal and plant hosts.
- Siderophores and siderophore uptake show great potential as biomarkers and for imaging of fungal infections.
- The siderophore system might allow fungal-specific drug delivery by a Trojan horse approach in which toxic compounds are conjugated to siderophores for selective import by fungal cells.

## Abbreviations

^68^Ga: Gallium-68
^89^Zr: Zirkonium-89
CBC: CCAAT-binding complex
CoA: coenzyme A
CRR: cysteine-rich region
CT: computer tomography
FC: ferricrocin
FsC: fusarinine C
NIS: NRPS-independent synthetase
NRPS: nonribosomal peptide synthetase
PET: positron emission tomography
RIA: reductive iron assimilation
SIA: siderophore-mediated iron acquisition
SIT: siderophore iron transporter
TAFC: triacetylfusarinine C

## References

[1] Hyde K.D., Xu J., Rapior S., Jeewon R., Lumyong S., Niego A.G.T. et al. (2019) The amazing potential of fungi: 50 ways we can exploit fungi industrially. Fungal Divers. 97, 1–136 10.1007/s13225-019-00430-9

[2] Meyer V., Basenko E.Y., Benz J.P., Braus G.H., Caddick M.X., Csukai M. et al. (2020) Growing a circular economy with fungal biotechnology: a white paper. Fungal Biol. Biotechnol. 7, 5 10.1186/s40694-020-00095-z 32280481 PMC7140391

[3] Fisher M.C., Gurr S.J., Cuomo C.A., Blehert D.S., Jin H., Stukenbrock E.H. et al. (2020) Threats posed by the fungal kingdom to humans, wildlife, and agriculture. MBio 11, e00449–e00520 10.1128/mBio.00449-20 32371596 PMC7403777

[4] Clayton D.D. (1969) The origin of the elements. Phys. Today 22, 28–36 10.1063/1.3035572

[5] Misslinger M., Hortschansky P., Brakhage A.A. and Haas H. (2021) Fungal iron homeostasis with a focus on *Aspergillus fumigatus*. Biochim. Biophys. Acta Mol. Cell Res. 1868, 118885 10.1016/j.bbamcr.2020.118885 33045305

[6] Haber F., Weiss J. and Pope W.J. (1997) The catalytic decomposition of hydrogen peroxide by iron salts. Proc. R. Soc. Lond. A - Math. Phys. Sci. 147, 332–351

[7] Halliwell B. and Gutteridge J.M. (1984) Role of iron in oxygen radical reactions. Methods Enzymol. 105, 47–56 10.1016/S0076-6879(84)05007-2 6203010

[8] Kornitzer D. and Roy U. (2020) Pathways of heme utilization in fungi. Biochim. Biophys. Acta Mol. Cell Res. 1867, 118817 10.1016/j.bbamcr.2020.118817 32777371

[9] Hider R.C. and Kong X. (2010) Chemistry and biology of siderophores. Nat. Prod. Rep. 27, 637–657 10.1039/b906679a 20376388

[10] Schrettl M., Bignell E., Kragl C., Joechl C., Rogers T., Arst H.N. et al. (2004) Siderophore biosynthesis but not reductive iron assimilation is essential for *Aspergillus fumigatus* virulence. J. Exp. Med. 200, 1213–1219 10.1084/jem.20041242 15504822 PMC2211866

[11] Gsaller F., Eisendle M., Lechner B.E., Schrettl M., Lindner H., Müller D. et al. (2012) The interplay between vacuolar and siderophore-mediated iron storage in *Aspergillus fumigatus*. Metallomics 4, 1262–1270 10.1039/c2mt20179h 23151814

[12] Thieken A. and Winkelmann G. (1992) Rhizoferrin: a complexone type siderophore of the mocorales and entomophthorales (Zygomycetes). FEMS Microbiol. Lett. 94, 37–41 10.1111/j.1574-6968.1992.tb05285.x 1387861

[13] Haselwandter K., Haas H., Häninger G. and Winkelmann G. (2020) Siderophores in plant root tissue: tagetes patula nana colonized by the arbuscular mycorrhizal fungus *Gigaspora margarita*. Biometals 33, 137–146 10.1007/s10534-020-00238-0 32363469

[14] Carroll C.S., Grieve C.L., Murugathasan I., Bennet A.J., Czekster C.M., Liu H. et al. (2017) The rhizoferrin biosynthetic gene in the fungal pathogen Rhizopus delemar is a novel member of the NIS gene family. Int. J. Biochem. Cell Biol. 89, 136–146 10.1016/j.biocel.2017.06.005 28610916

[15] Renshaw J.C., Robson G.D., Trinci A.P.J., Wiebe M.G., Livens F.R., Collison D. et al. (2002) Fungal siderophores: structures, functions and applications. Mycol. Res. 106, 1123–1142 10.1017/S0953756202006548

[16] Khan A., Singh P. and Srivastava A. (2018) Synthesis, nature and utility of universal iron chelator - Siderophore: a review. Microbiol. Res. 212-213, 103–111 10.1016/j.micres.2017.10.012 29103733

[17] Kobayashi T., Nozoye T. and Nishizawa N.K. (2019) Iron transport and its regulation in plants. Free Radic. Biol. Med. 133, 11–20 10.1016/j.freeradbiomed.2018.10.439 30385345

[18] Schrettl M., Bignell E., Kragl C., Sabiha Y., Loss O., Eisendle M. et al. (2007) Distinct roles for intra- and extracellular siderophores during *Aspergillus fumigatus* infection. PLoS Pathog. 3, e128 10.1371/journal.ppat.0030128 17845073 PMC1971116

[19] Aguiar M., Orasch T., Misslinger M., Dietl A.-M., Gsaller F. and Haas H. (2021) The siderophore transporters Sit1 and Sit2 are essential for utilization of ferrichrome-, ferrioxamine- and coprogen-type siderophores in *Aspergillus fumigatus*. J. Fungi (Basel) 7, 768 10.3390/jof7090768 34575806 PMC8470733

[20] Wallner A., Blatzer M., Schrettl M., Sarg B., Lindner H. and Haas H. (2009) Ferricrocin, a siderophore involved in intra- and transcellular iron distribution in *Aspergillus fumigatus*. Appl. Environ. Microbiol. 75, 4194–4196 10.1128/AEM.00479-09 19376908 PMC2698346

[21] Eisendle M., Schrettl M., Kragl C., Müller D., Illmer P. and Haas H. (2006) The intracellular siderophore ferricrocin is involved in iron storage, oxidative-stress resistance, germination, and sexual development in *Aspergillus nidulans*. Eukaryot. Cell 5, 1596–1603 10.1128/EC.00057-06 17030991 PMC1595343

[22] Happacher I., Aguiar M., Alilou M., Abt B., Baltussen T.J.H., Decristoforo C. et al. (2023) The siderophore ferricrocin mediates iron acquisition in *Aspergillus fumigatus*. Microbiol. Spectr. 0, e00496–e00523 10.1128/spectrum.00496-23 PMC10269809 37199664

[23] Yasmin S., Alcazar-Fuoli L., Gründlinger M., Puempel T., Cairns T., Blatzer M. et al. (2012) Mevalonate governs interdependency of ergosterol and siderophore biosyntheses in the fungal pathogen *Aspergillus fumigatus*. Proc. Natl. Acad. Sci. U.S.A. 109, E497–E504 10.1073/pnas.1106399108 22106303 PMC3286978

[24] Haas H. (2014) Fungal siderophore metabolism with a focus on *Aspergillus fumigatus*. Nat. Prod. Rep. 31, 1266–1276 10.1039/C4NP00071D 25140791 PMC4162504

[25] Gründlinger M., Yasmin S., Lechner B.E., Geley S., Schrettl M., Hynes M. et al. (2013) Fungal siderophore biosynthesis is partially localized in peroxisomes. Mol. Microbiol. 88, 862–875 10.1111/mmi.12225 23617799 PMC3709128

[26] Oide S., Moeder W., Krasnoff S., Gibson D., Haas H., Yoshioka K. et al. (2006) NPS6, encoding a nonribosomal peptide synthetase involved in siderophore-mediated iron metabolism, is a conserved virulence determinant of plant pathogenic ascomycetes. Plant Cell 18, 2836–2853 10.1105/tpc.106.045633 17056706 PMC1626607

[27] Hai Y., Jenner M. and Tang Y. (2020) Fungal siderophore biosynthesis catalysed by an iterative nonribosomal peptide synthetase. Chem. Sci. 11, 11525–11530 10.1039/D0SC03627G 34094397 PMC8162485

[28] Blatzer M., Schrettl M., Sarg B., Lindner H.H., Pfaller K. and Haas H. (2011) SidL, an *Aspergillus fumigatus* Transacetylase Involved in Biosynthesis of the Siderophores Ferricrocin and Hydroxyferricrocin ▿. Appl. Environ. Microbiol. 77, 4959–4966 10.1128/AEM.00182-11 21622789 PMC3147410

[29] Johnson L.J., Koulman A., Christensen M., Lane G.A., Fraser K., Forester N. et al. (2013) An extracellular siderophore is required to maintain the mutualistic interaction of Epichloë festucae with Lolium perenne. PLoS Pathog. 9, e1003332 10.1371/journal.ppat.1003332 23658520 PMC3642064

[30] Haas H., Eisendle M. and Turgeon B.G. (2008) Siderophores in fungal physiology and virulence. Annu. Rev. Phytopathol. 46, 149–187 10.1146/annurev.phyto.45.062806.094338 18680426

[31] Philpott C.C., Leidgens S. and Frey A.G. (2012) Metabolic remodeling in iron-deficient fungi. Biochim. Biophys. Acta 1823, 1509–1520 10.1016/j.bbamcr.2012.01.012 22306284 PMC3348335

[32] Park Y.-S., Kim J.-Y. and Yun C.-W. (2016) Identification of ferrichrome- and ferrioxamine B-mediated iron uptake by *Aspergillus fumigatus*. Biochem. J. 473, 1203–1213 10.1042/BCJ20160066 26929401

[33] Aguiar M., Orasch T., Shadkchan Y., Caballero P., Pfister J., Sastré-Velásquez L.E. et al. (2022) Uptake of the siderophore triacetylfusarinine C, but not fusarinine C, is crucial for virulence of *Aspergillus fumigatus*. MBio 0, e02192–e02222 10.1128/mbio.02192-22 PMC9600649 36125294

[34] Dietl A.-M., Misslinger M., Aguiar M.M., Ivashov V., Teis D., Pfister J. et al. (2019) The siderophore transporter Sit1 determines susceptibility to the antifungal VL-2397. Antimicrob. Agents Chemother. 63, e00807–e00919 10.1128/AAC.00807-19 31405865 PMC6761561

[35] Misslinger M., Petrik M., Pfister J., Hubmann I., Bendova K., Decristoforo C. et al. (2021) Desferrioxamine B-mediated pre-clinical in vivo imaging of infection by the mold fungus *Aspergillus fumigatus*. J. Fungi (Basel) 7, 734 10.3390/jof7090734 34575772 PMC8472378

[36] Haas H., Schoeser M., Lesuisse E., Ernst J.F., Parson W., Abt B. et al. (2003) Characterization of the *Aspergillus nidulans* transporters for the siderophores enterobactin and triacetylfusarinine C. Biochem. J. 371, 505–513 10.1042/bj20021685 12487628 PMC1223275

[37] Park Y.-S., Kim T.-H., Chang H.-I., Sung H.-C. and Yun C.-W. (2006) Cellular iron utilization is regulated by putative siderophore transporter FgSit1 not by free iron transporter in *Fusarium graminearum*. Biochem. Biophys. Res. Commun. 345, 1634–1642 10.1016/j.bbrc.2006.05.071 16750173

[38] Tangen K.L., Jung W.H., Sham A.P., Lian T. and Kronstad J.W. (2007) The iron- and cAMP-regulated gene SIT1 influences ferrioxamine B utilization, melanization and cell wall structure in *Cryptococcus neoformans*. Microbiology (Reading) 153, 29–41 10.1099/mic.0.2006/000927-0 17185532

[39] Mulvihill E.D., Moloney N.M., Owens R.A., Dolan S.K., Russell L. and Doyle S. (2017) Functional investigation of iron-responsive microsomal proteins, including MirC, in *Aspergillus fumigatus*. Front. Microbiol. 8, 418 10.3389/fmicb.2017.00418 28367141 PMC5355445

[40] Dhaoui M., Auchère F., Blaiseau P.-L., Lesuisse E., Landoulsi A., Camadro J.-M. et al. (2011) Gex1 is a yeast glutathione exchanger that interferes with pH and redox homeostasis. Mol. Biol. Cell 22, 2054–2067 10.1091/mbc.e10-11-0906 21490148 PMC3113770

[41] Normant V., Mourer T. and Labbé S. (2018) The major facilitator transporter Str3 is required for low-affinity heme acquisition in *Schizosaccharomyces pombe*. J. Biol. Chem. 293, 6349–6362 10.1074/jbc.RA118.002132 29549126 PMC5925805

[42] Plante S. and Labbé S. (2019) Spore germination requires ferrichrome biosynthesis and the siderophore transporter Str1 in *Schizosaccharomyces pombe*. Genetics 211, 893–911 10.1534/genetics.118.301843 30647069 PMC6404258

[43] Kragl C., Schrettl M., Abt B., Sarg B., Lindner H.H. and Haas H. (2007) EstB-mediated hydrolysis of the siderophore triacetylfusarinine C optimizes iron uptake of *Aspergillus fumigatus*. Eukaryot. Cell. 6, 1278–1285 10.1128/EC.00066-07 17586718 PMC1951140

[44] Gründlinger M., Gsaller F., Schrettl M., Lindner H. and Haas H. (2013) *Aspergillus fumigatus* SidJ mediates intracellular siderophore hydrolysis. Appl. Environ. Microbiol. 79, 7534–7536 10.1128/AEM.01285-13 24038704 PMC3837724

[45] Ecker F., Haas H., Groll M. and Huber E.M. (2018) Iron scavenging in *Aspergillus* species: structural and biochemical insights into fungal siderophore esterases. Angew. Chem. Int. Ed. 57, 14624–14629 10.1002/anie.201807093 30070018

[46] Cain T.J. and Smith A.T. (2021) Ferric iron reductases and their contribution to unicellular ferrous iron uptake. J. Inorg. Biochem. 218, 111407 10.1016/j.jinorgbio.2021.111407 33684686 PMC8035299

[47] Misslinger M., Scheven M.T., Hortschansky P., López-Berges M.S., Heiss K., Beckmann N. et al. (2019) The monothiol glutaredoxin GrxD is essential for sensing iron starvation in *Aspergillus fumigatus*. PLos Genet. 15, e1008379 10.1371/journal.pgen.1008379 31525190 PMC6762210

[48] Schrettl M., Beckmann N., Varga J., Heinekamp T., Jacobsen I.D., Jöchl C. et al. (2010) HapX-mediated adaption to iron starvation is crucial for virulence of *Aspergillus fumigatus*. PLoS Pathog. 6, e1001124 10.1371/journal.ppat.1001124 20941352 PMC2947994

[49] Hortschansky P., Eisendle M., Al-Abdallah Q., Schmidt A.D., Bergmann S., Thön M. et al. (2007) Interaction of HapX with the CCAAT-binding complex-a novel mechanism of gene regulation by iron. EMBO J. 26, 3157–3168 10.1038/sj.emboj.7601752 17568774 PMC1914100

[50] Misslinger M., Lechner B.E., Bacher K. and Haas H. (2018) Iron-sensing is governed by mitochondrial, not by cytosolic iron-sulfur cluster biogenesis in *Aspergillus fumigatus*†. Metallomics 10, 1687–1700 10.1039/C8MT00263K 30395137 PMC6250123

[51] Kohlhaw G.B. (2003) Leucine biosynthesis in fungi: entering metabolism through the back door. Microbiol. Mol. Biol. Rev. 67, 1–15 10.1128/MMBR.67.1.1-15.2003 12626680 PMC150519

[52] Long N., Orasch T., Zhang S., Gao L., Xu X., Hortschansky P. et al. (2018) The Zn2Cys6-type transcription factor LeuB cross-links regulation of leucine biosynthesis and iron acquisition in *Aspergillus fumigatus*. PLos Genet. 14, e1007762 10.1371/journal.pgen.1007762 30365497 PMC6221358

[53] Orasch T., Dietl A.-M., Shadkchan Y., Binder U., Bauer I., Lass-Flörl C. et al. (2019) The leucine biosynthetic pathway is crucial for adaptation to iron starvation and virulence in *Aspergillus fumigatus*. Virulence 10, 925–934 10.1080/21505594.2019.1682760 31694453 PMC6844326

[54] Blatzer M., Barker B.M., Willger S.D., Beckmann N., Blosser S.J., Cornish E.J. et al. (2011) SREBP coordinates iron and ergosterol homeostasis to mediate triazole drug and hypoxia responses in the human fungal pathogen *Aspergillus fumigatus*. PLos Genet. 7, e1002374 10.1371/journal.pgen.1002374 22144905 PMC3228822

[55] Yap A., Volz R., Paul S., Moye-Rowley W.S. and Haas H. (2023) Regulation of high-affinity iron acquisition, including acquisition mediated by the iron permease FtrA, is coordinated by AtrR, SrbA, and SreA in *Aspergillus fumigatus*. MBio 0, e00757–e00823 10.1128/mbio.00757-23 PMC10294635 37093084

[56] Chung D., Haas H. and Cramer R.A. (2012) Coordination of hypoxia adaptation and iron homeostasis in human pathogenic fungi. Front. Microbiol. 3, 381 10.3389/fmicb.2012.00381 23133438 PMC3490150

[57] Hagiwara D., Miura D., Shimizu K., Paul S., Ohba A., Gonoi T. et al. (2017) A novel Zn2-Cys6 transcription factor AtrR plays a key role in an azole resistance mechanism of *Aspergillus fumigatus* by co-regulating cyp51A and cdr1B expressions. PLoS Pathog. 13, e1006096 10.1371/journal.ppat.1006096 28052140 PMC5215518

[58] Vödisch M., Scherlach K., Winkler R., Hertweck C., Braun H.-P., Roth M. et al. (2011) Analysis of the *Aspergillus fumigatus* proteome reveals metabolic changes and the activation of the pseurotin A biosynthesis gene cluster in response to hypoxia. J. Proteome Res. 10, 2508–2524 10.1021/pr1012812 21388144 PMC3091480

[59] Ganz T. (2009) Iron in innate immunity: starve the invaders. Curr. Opin. Immunol. 21, 63–67 10.1016/j.coi.2009.01.011 19231148 PMC2668730

[60] Nairz M. and Weiss G. (2020) Iron in infection and immunity. Mol. Aspects Med. 75, 100864 10.1016/j.mam.2020.100864 32461004

[61] Matthaiou E.I., Sass G., Stevens D.A. and Hsu J.L. (2018) Iron: an essential nutrient for *Aspergillus fumigatus* and a fulcrum for pathogenesis. Curr. Opin. Infect. Dis. 31, 506 10.1097/QCO.0000000000000487 30379731 PMC6579532

[62] Hsu J.L., Manouvakhova O.V., Clemons K.V., Inayathullah M., Tu A.B., Sobel R.A. et al. (2018) Microhemorrhage-associated tissue iron enhances the risk for *Aspergillus fumigatus* invasion in a mouse model of airway transplantation. Sci. Transl. Med. 10, eaag2616 10.1126/scitranslmed.aag2616 29467298 PMC5841257

[63] Petzer V., Wermke M., Tymoszuk P., Wolf D., Seifert M., Ovaçin R. et al. (2019) Enhanced labile plasma iron in hematopoietic stem cell transplanted patients promotes *Aspergillus* outgrowth. Blood Adv. 3, 1695–1700 10.1182/bloodadvances.2019000043 31167821 PMC6560355

[64] Van Cutsem J. and Boelaert J.R. (1989) Effects of deferoxamine, feroxamine and iron on experimental mucormycosis (zygomycosis). Kidney Int. 36, 1061–1068 10.1038/ki.1989.301 2601256

[65] Boelaert J.R., de Locht M., Cutsem J.V., Kerrels V., Cantinieaux B., Verdonck A. et al. (1993) Mucormycosis during deferoxamine therapy is a siderophore-mediated infection. In vitro and in vivo animal studies. J. Clin. Invest. 91, 1979–1986 10.1172/JCI116419 8486769 PMC288195

[66] Mei B., Budde A.D. and Leong S.A. (1993) sid1, a gene initiating siderophore biosynthesis in Ustilago maydis: molecular characterization, regulation by iron, and role in phytopathogenicity. Proc. Natl. Acad. Sci. U.S.A. 90, 903–907 10.1073/pnas.90.3.903 8430103 PMC45778

[67] Greenshields D.L., Liu G., Feng J., Selvaraj G. and Wei Y. (2007) The siderophore biosynthetic gene SID1, but not the ferroxidase gene FET3, is required for full Fusarium graminearum virulence. Mol. Plant Pathol. 8, 411–421 10.1111/j.1364-3703.2007.00401.x 20507510

[68] Park Y.-S., Choi I.-D., Kang C.-M., Ham M.-S., Kim J.-H., Kim T.-H. et al. (2006) Functional identification of high-affinity iron permeases from Fusarium graminearum. Fungal Genet. Biol. 43, 273–282 10.1016/j.fgb.2005.12.005 16464625

[69] Voß B., Kirschhöfer F., Brenner-Weiß G. and Fischer R. (2020) Alternaria alternata uses two siderophore systems for iron acquisition. Sci. Rep. 10, 3587 10.1038/s41598-020-60468-7 32107432 PMC7046739

[70] Albarouki E., Schafferer L., Ye F., von Wirén N., Haas H. and Deising H.B. (2014) Biotrophy-specific downregulation of siderophore biosynthesis in *Colletotrichum graminicola* is required for modulation of immune responses of maize. Mol. Microbiol. 92, 338–355 10.1111/mmi.12561 24674132 PMC4235341

[71] Eichhorn H., Lessing F., Winterberg B., Schirawski J., Kämper J., Müller P. et al. (2006) A ferroxidation/permeation iron uptake system is required for virulence in *Ustilago maydis*. Plant Cell 18, 3332–3345 10.1105/tpc.106.043588 17138696 PMC1693961

[72] Aznar A. and Dellagi A. (2015) New insights into the role of siderophores as triggers of plant immunity: what can we learn from animals? J. Exp. Bot. 66, 3001–3010 10.1093/jxb/erv155 25934986

[73] Boughammoura A., Franza T., Dellagi A., Roux C., Matzanke-Markstein B. and Expert D. (2007) Ferritins, bacterial virulence and plant defence. Biometals 20, 347–353 10.1007/s10534-006-9069-0 17216356

[74] Expert D. (2006) Genetic regulation of iron in *Erwinia chrysanthemi* as pertains to bacterial virulence In Iron Nutrition in Plants and Rhizospheric Microorganisms (Barton L.L. and Abadia J. eds.) pp. 215–227. Springer, Dordrecht 10.1007/1-4020-4743-6_10

[75] Alejandre-Castañeda V., Patiño-Medina J.A., Valle-Maldonado M.I., Nuñez-Anita R.E., Santoyo G., Castro-Cerritos K.V. et al. (2022) Secretion of the siderophore rhizoferrin is regulated by the cAMP-PKA pathway and is involved in the virulence of *Mucor lusitanicus*. Sci. Rep. 12, 10649 10.1038/s41598-022-14515-0 35739200 PMC9226013

[76] Dietl A.-M., Binder U., Bauer I., Shadkchan Y., Osherov N. and Haas H. (2020) Arginine auxotrophy affects siderophore biosynthesis and attenuates virulence of *Aspergillus fumigatus*. Genes 11, 423 10.3390/genes11040423 32326414 PMC7231135

[77] Dietl A.-M., Meir Z., Shadkchan Y., Osherov N. and Haas H. (2018) Riboflavin and pantothenic acid biosynthesis are crucial for iron homeostasis and virulence in the pathogenic mold *Aspergillus fumigatus*. Virulence 9, 1036–1049 10.1080/21505594.2018.1482181 30052132 PMC6068542

[78] Forester N.T., Lane G.A., McKenzie C.M., Lamont I.L. and Johnson L.J. (2019) The role of SreA-mediated iron regulation in maintaining Epichloë festucae-Lolium perenne Symbioses. MPMI 32, 1324–1335 10.1094/MPMI-03-19-0060-R 31107632

[79] Santus W., Rana A.P., Devlin J.R., Kiernan K.A., Jacob C.C., Tjokrosurjo J. et al. (2022) Mycobiota and diet-derived fungal xenosiderophores promote Salmonella gastrointestinal colonization. Nat. Microbiol. 7, 2025–2038 10.1038/s41564-022-01267-w 36411353 PMC11981548

[80] Braun V., Günter K. and Hantke K. (1991) Transport of iron across the outer membrane. Biol. Metals 4, 14–22 10.1007/BF01135552 1854585

[81] Grinter R. and Lithgow T. (2019) Determination of the molecular basis for coprogen import by Gram-negative bacteria. IUCrJ. 6, 401–411 10.1107/S2052252519002926 31098021 PMC6503915

[82] Sass G., Nazik H., Penner J., Shah H., Ansari S.R., Clemons K.V. et al. (2017) Studies of *Pseudomonas aeruginos*a mutants indicate pyoverdine as the central factor in inhibition of *Aspergillus fumigatus* biofilm. J. Bacteriol. 200, e00345–e00417 10.1093/ofid/ofx163.137 29038255 PMC5717155

[83] Sass G., Ansari S.R., Dietl A.-M., Déziel E., Haas H. and Stevens D.A. (2019) Intermicrobial interaction: *Aspergillus fumigatus* siderophores protect against competition by *Pseudomonas aeruginosa*. PloS ONE 14, e0216085 10.1371/journal.pone.0216085 31067259 PMC6505954

[84] Anke H. (1977) Metabolic products of microoorganisms. 163. Desferritriacetylfusigen, an antibiotic from *Aspergillus deflectus*. J. Antibiot. (Tokyo) 30, 125–128 10.7164/antibiotics.30.125 849914

[85] Budde A.D. and Leong S.A. (1989) Characterization of siderophores from *Ustilago maydis*. Mycopathologia 108, 125–133 10.1007/BF00436063 2531844

[86] Thanh V.N., Van Dyk M.S. and Wingfield M.J. (2002) Debaryomyces mycophilus sp. nov., a siderophore-dependent yeast isolated from woodlice. FEMS Yeast Res. 2, 415–427 12702293 10.1111/j.1567-1364.2002.tb00112.x

[87] Beguin H. (2010) Tritirachium egenum, a thiamine- and siderophore-auxotrophic fungal species isolated from a Penicillium rugulosum. FEMS Microbiol. Ecol. 74, 165–173 10.1111/j.1574-6941.2010.00929.x 20618858

[88] Bernhardt P.V. (2007) Coordination chemistry and biology of chelators for the treatment of iron overload disorders. Dalton Trans. 3214–3220 10.1039/b708133b 17893764

[89] Petrik M., Zhai C., Haas H. and Decristoforo C. (2017) Siderophores for molecular imaging applications. Clin. Transl. Imaging 5, 15–27 10.1007/s40336-016-0211-x 28138436 PMC5269471

[90] Heskamp S., Raavé R., Boerman O., Rijpkema M., Goncalves V. and Denat F. (2017) 89Zr-immuno-positron emission tomography in oncology: state-of-the-art 89Zr radiochemistry. Bioconjug. Chem. 28, 2211–2223 10.1021/acs.bioconjchem.7b00325 28767228 PMC5609224

[91] Melendez-Alafort L., Ferro-Flores G., De Nardo L., Ocampo-García B. and Bolzati C. (2023) Zirconium immune-complexes for PET molecular imaging: current status and prospects. Coord. Chem. Rev. 479, 215005 10.1016/j.ccr.2022.215005

[92] Knetsch P.A., Zhai C., Rangger C., Blatzer M., Haas H., Kaeopookum P. et al. (2015) [(68)Ga]FSC-(RGD)3 a trimeric RGD peptide for imaging αvβ3 integrin expression based on a novel siderophore derived chelating scaffold-synthesis and evaluation. Nucl. Med. Biol. 42, 115–122 10.1016/j.nucmedbio.2014.10.001 25459110 PMC4289911

[93] Zhai C., Summer D., Rangger C., Franssen G.M., Laverman P., Haas H. et al. (2015) Novel bifunctional cyclic chelator for (89)Zr labeling-radiolabeling and targeting properties of RGD conjugates. Mol. Pharm. 12, 2142–2150 10.1021/acs.molpharmaceut.5b00128 25941834 PMC4453016

[94] Summer D., Rangger C., Klingler M., Laverman P., Franssen G.M., Lechner B.E. et al. (2018) Exploiting the concept of multivalency with 68Ga- and 89Zr-labelled fusarinine C-minigastrin bioconjugates for targeting CCK2R expression. Contrast Media Mol. Imaging 2018, 3171794 10.1155/2018/3171794 29849512 PMC5914118

[95] Summer D., Grossrubatscher L., Petrik M., Michalcikova T., Novy Z., Rangger C. et al. (2017) Developing targeted hybrid imaging probes by chelator scaffolding. Bioconjug. Chem. 28, 1722–1733 10.1021/acs.bioconjchem.7b00182 28462989 PMC5481817

[96] Summer D., Mayr S., Petrik M., Rangger C., Schoeler K., Vieider L. et al. (2018) Pretargeted Imaging with Gallium-68-Improving the Binding Capability by Increasing the Number of Tetrazine Motifs. Pharmaceuticals (Basel) 11, 102 10.3390/ph11040102 30314332 PMC6316846

[97] Summer D., Garousi J., Oroujeni M., Mitran B., Andersson K.G., Vorobyeva A. et al. (2018) Cyclic versus noncyclic chelating scaffold for 89Zr-labeled ZEGFR:2377 affibody bioconjugates targeting epidermal growth factor receptor overexpression. Mol. Pharm. 15, 175–185 10.1021/acs.molpharmaceut.7b00787 29160082 PMC5751887

[98] Latgé J.-P. and Chamilos G. (2019) Aspergillus fumigatus and Aspergillosis in 2019. Clin. Microbiol. Rev. 33, e00140–e00218 10.1128/CMR.00140-18 31722890 PMC6860006

[99] Orasch T., Prattes J., Faserl K., Eigl S., Düttmann W., Lindner H. et al. (2017) Bronchoalveolar lavage triacetylfusarinine C (TAFC) determination for diagnosis of invasive pulmonary aspergillosis in patients with hematological malignancies. J. Infect. 75, 370–373 10.1016/j.jinf.2017.05.014 28576596 PMC5757784

[100] Hoenigl M., Orasch T., Faserl K., Prattes J., Loeffler J., Springer J. et al. (2019) Triacetylfusarinine C: a urine biomarker for diagnosis of invasive aspergillosis. J. Infect. 78, 150–157 10.1016/j.jinf.2018.09.006 30267801 PMC6361682

[101] Kriegl L., Havlicek V., Dichtl K., Egger M. and Hoenigl M. (2022) Siderophores: a potential role as a diagnostic for invasive fungal disease. Curr. Opin. Infect. Dis. 35, 485–492 10.1097/QCO.0000000000000862 35942851

[102] Petrik M., Haas H., Schrettl M., Helbok A., Blatzer M. and Decristoforo C. (2012) *In vitro* and *in vivo* evaluation of selected 68Ga-siderophores for infection imaging. Nucl. Med. Biol. 39, 361–369 10.1016/j.nucmedbio.2011.09.012 22172389 PMC3314960

[103] Moloney N.M., Larkin A., Xu L., Fitzpatrick D.A., Crean H.L., Walshe K. et al. (2021) Generation and characterisation of a semi-synthetic siderophore-immunogen conjugate and a derivative recombinant triacetylfusarinine C-specific monoclonal antibody with fungal diagnostic application. Anal. Biochem. 632, 114384 10.1016/j.ab.2021.114384 34543643

[104] Pahlow S., Orasch T., Žukovskaja O., Bocklitz T., Haas H. and Weber K. (2020) Rapid detection of the aspergillosis biomarker triacetylfusarinine C using interference-enhanced Raman spectroscopy. Anal. Bioanal. Chem. 412, 6351–6360 10.1007/s00216-020-02571-2 32170382 PMC7442771

[105] Petrik M., Haas H., Dobrozemsky G., Lass-Flörl C., Helbok A., Blatzer M. et al. (2010) 68Ga-siderophores for PET imaging of invasive pulmonary aspergillosis: proof of principle. J. Nucl. Med. 51, 639–645 10.2967/jnumed.109.072462 20351354 PMC2992174

[106] Petrik M., Franssen G.M., Haas H., Laverman P., Hörtnagl C., Schrettl M. et al. (2012) Preclinical evaluation of two 68Ga-siderophores as potential radiopharmaceuticals for *Aspergillus fumigatus* infection imaging. Eur. J. Nucl. Med. Mol. Imaging 39, 1175–1183 10.1007/s00259-012-2110-3 22526953 PMC3369139

[107] Petrik M., Haas H., Laverman P., Schrettl M., Franssen G.M., Blatzer M. et al. (2014) 68Ga-triacetylfusarinine C and 68Ga-ferrioxamine E for Aspergillus infection imaging: uptake specificity in various microorganisms. Mol. Imaging Biol. 16, 102–108 10.1007/s11307-013-0654-7 23818006 PMC3823598

[108] Pfister J., Summer D., Petrik M., Khoylou M., Lichius A., Kaeopookum P. et al. (2020) Hybrid Imaging of *Aspergillus fumigatus* pulmonary infection with fluorescent, 68Ga-labelled siderophores. Biomolecules 10, 168 10.3390/biom10020168 31979017 PMC7072563

[109] Pfister J., Lichius A., Summer D., Haas H., Kanagasundaram T., Kopka K. et al. (2020) Live-cell imaging with *Aspergillus fumigatus*-specific fluorescent siderophore conjugates. Sci. Rep. 10, 15519 10.1038/s41598-020-72452-2 32968138 PMC7511942

[110] Miller M.J., Zhu H., Xu Y., Wu C., Walz A.J., Vergne A. et al. (2009) Utilization of microbial iron assimilation processes for the development of new antibiotics and inspiration for the design of new anticancer agents. Biometals 22, 61–75 10.1007/s10534-008-9185-0 19130268 PMC4066965

[111] Gumienna-Kontecka E. and Carver P.L. (2019) Building a trojan horse: siderophore-drug conjugates for the treatment of infectious diseases. Met. Ions Life Sci. 19, , https://books/9783110527872/9783110527872-013/9783110527872-013.xml 10.1515/9783110527872-007 30855108

[112] Pfister J., Petrik M., Bendova K., Matuszczak B., Binder U., Misslinger M. et al. (2021) Antifungal siderophore conjugates for theranostic applications in invasive pulmonary aspergillosis using low-molecular TAFC scaffolds. J. Fungi (Basel) 7, 558 10.3390/jof7070558 34356941 PMC8304796

[113] Negash K.H., Norris J.K.S. and Hodgkinson J.T. (2019) Siderophore-antibiotic conjugate design: new drugs for bad bugs? Molecules 24, 3314 10.3390/molecules24183314 31514464 PMC6767078

[114] Martín del Campo J.S., Vogelaar N., Tolani K., Kizjakina K., Harich K. and Sobrado P. (2016) Inhibition of the flavin-dependent monooxygenase siderophore A (SidA) blocks siderophore biosynthesis and *Aspergillus fumigatus* growth. ACS Chem. Biol. 11, 3035–3042 10.1021/acschembio.6b00666 27588426

[115] Leal S.M. Jr, Roy S., Vareechon C., deJesus Carrion S., Clark H., Lopez-Berges M.S. et al. (2013) Targeting iron acquisition blocks infection with the fungal pathogens *Aspergillus fumigatus* and *Fusarium oxysporum*. PLoS Pathog. 9, e1003436 10.1371/journal.ppat.1003436 23853581 PMC3708856

